# Augmentation of DMLS Biomimetic Dental Implants with Weight-Bearing Strut to Balance of Biologic and Mechanical Demands: From Bench to Animal

**DOI:** 10.3390/ma12010164

**Published:** 2019-01-07

**Authors:** Jenny Zwei-Chieng Chang, Pei-I Tsai, Mark Yen-Ping Kuo, Jui-Sheng Sun, San-Yuan Chen, Hsin-Hsin Shen

**Affiliations:** 1 School of Dentistry, College of Medicine, National Taiwan University, Taipei 10051, Taiwan; jennyzc@ms3.hinet.net; 2 Department of Materials Science and Engineering, National Chiao-Tung University, Hsinchu 30010, Taiwan; peiyi@itri.org.tw (P.-I.T.); sanyuanchen@mail.nctu.edu.tw (S.-Y.C.); 3 Biomedical Technology and Device Research Laboratories, Industrial Technology Research Institute, Hsinchu 31040, Taiwan; shenhsin@itri.org.tw; 4 Department of Orthopedic Surgery, College of Medicine, National Taiwan University, Taipei 10002, Taiwan

**Keywords:** direct metal laser sintering, biomimetic, porous titanium, Ti_6_Al_4_V, 3D-printing

## Abstract

A mismatch of elastic modulus values could result in undesirable bone resorption around the dental implant. The objective of this study was to optimize direct metal laser sintering (DMLS)-manufactured Ti_6_Al_4_V dental implants’ design, minimize elastic mismatch, allow for maximal bone ingrowth, and improve long-term fixation of the implant. In this study, DMLS dental implants with different morphological characteristics were fabricated. Three-point bending, torsional, and stability tests were performed to compare the mechanical properties of different designs. Improvement of the weaker design was attempted by augmentation with a longitudinal 3D-printed strut. The osseointegrative properties were evaluated. The results showed that the increase in porosity decreased the mechanical properties, while augmentation with a longitudinal weight-bearing strut can improve mechanical strength. Maximal alkaline phosphatase gene expression of MG63 cells attained on 60% porosity Ti_6_Al_4_V discs. In vivo experiments showed good incorporation of bone into the porous scaffolds of the DMLS dental implant, resulting in a higher pull-out strength. In summary, we introduced a new design concept by augmenting the implant with a longitudinal weight-bearing strut to achieve the ideal combination of high strength and low elastic modulus; our results showed that there is a chance to reach the balance of both biologic and mechanical demands.

## 1. Introduction

Several factors influencing the osseointegration of dental implants include biocompatibility of implant material, implant surface and design, healthy implant bed and good bone quality, surgical technique, adequate healing phase, and loading conditions [1]. Among these, the implant material, surface topography, and design are factors that may be controlled during the manufacturing process of the dental implant. The vast majority of currently available dental implants are made of commercially pure (grade 4) titanium. Because of increased failure with pure titanium implants in areas subjected to high wear or tensile strength, titanium alloy implants have emerged as an alternative choice, owing to their enhanced mechanical characteristics [2]. The reason for titanium and its alloys being the materials of choice for dental implants is their excellent biocompatibility. Among the titanium alloys for biomedical applications, the system titanium-aluminum-vanadium (Ti_6_Al_4_V; grade V titanium) is commonly used in orthopedic and dental implants. Adding aluminum and vanadium to titanium significantly increases the strength, ductility, and fatigue resistance of the material [2].

The elastic modulus is an important measure for biomechanical interaction between the implant and the bone. Although Ti_6_Al_4_V exhibits superior mechanical properties and comparable biocompatibility to conventional pure titanium, it presents a slightly higher elastic modulus. In the case of orthopedic prostheses, a mismatch of elastic modulus values could result in stress shielding that leads to undesirable bone resorption around the implant and subsequent aseptic loosening of the implant [3]. The reported Young’s modulus values in the literature range between 6.9–25 GPa for bone, 103–107 GPa for commercially pure titanium, and 114–120 GPa for Ti_6_Al_4_V [4]. It is very likely that the stress shielding phenomenon in conjunction with the complicated oral environment may contribute to the progressive peri-implant bone loss beyond the initial physiologic bone remodeling. An ideal dental implant is expected to possess a combination of high strength and low elastic modulus that make it strong enough to prevent deformation during insertion, and to withstand loading from tension, compression, bending, or torsion, to assure stability between the implant and prosthetic components, yet it must have low stiffness to avoid shielding the bone from stress. Unfortunately, these characteristics seem contradictory for solid metal materials. Fabrication of open-cell porous titanium alloy implants may be a good solution that reduces the effective elastic modulus to minimize the elastic mismatch, allowing for bone ingrowth inside the implant to enhance fixation [5].

Implant design/macrogeometry and the related surgical instrumentation technique/dimension are important parameters for attaining primary stability and successful osseointegration [6,7]. Studies investigating the effect of dental implant macrodesign on osseointegration mainly focus on design features, including implant size, body shape, thread design, crestal module, and microthreads [6,7]. Several methods have also been advocated to modify implant surface roughness for enhancing osseointegration [8]. Surface roughness may be divided into three levels, namely macro-, micro-, and nano-scale roughness [9]. Nowadays, commercially available dental implants are mostly fabricated by machining titanium rods, followed by modification techniques that produce a micro- or nano-rough implant surface on a high-density titanium structure. It is noteworthy that the macrogeometric design of an implant also increases the micro- and nano-level contribution to osseointegration [10]. Until recently, several methods have been developed for manufacturing porous titanium or titanium alloy implants to promote ingrowth of bone beyond the surface [4]. Direct metal laser sintering (DMLS) is a three-dimensional (3D) printing additive technique, in which a 3D porous structure may be built layer by layer using a high-powered laser beam to fuse powdered metal according to a predefined computer-assisted-design (CAD) file [11]. Recent studies have demonstrated the potential use of DMLS titanium alloy dental implants [11]. However, these DMLS dental implants are mostly designed with a very superficial porous, but non-scaffold surface, and a dense titanium core. As porosity and pore size of scaffolds for bone regeneration have been recognized to directly affect bone ingrowth [12], the objective of this study was to optimize DMLS-manufactured Ti_6_Al_4_V dental implant design, minimize elastic mismatch, allow maximal bone ingrowth, and improve long-term fixation of the implant.

## 2. Materials and Methods

### 2.1. Fabrication of Laser-Sintered Titanium Dental Implants

Seven types of laser-sintered dental implant specimens (4.3 mm in diameter; 10.0 mm in length) were produced using the EOSINT M 280 system (EOS GmbH, Munich, Germany). Briefly, the process computer sliced the predefined CAD file into a stack of thin slices. A uniform thin layer of Ti_6_Al_4_V powder (20–50 μm) was deposited using a moving wiper. The computer scanned the laser beam on the powder bed surface under a controlled hypoxia atmosphere (O_2_ < 10,000 ppm) to bond the loose powder together. The process was repeated, and by altering the shape of each scan layer, a part of arbitrary shape could be produced. All specimens were produced directly and fully automatically from 3D CAD data without any tooling.

### 2.2. Characterization of the Porous Biomimetic Ti_6_Al_4_V Dental Implants

The basic shapes of the dental implants were identical to the commercially available Screw-Vent^®^ Implant System (Zimmer Dental, Carlsbad, CA, USA) but with varying surface characteristics: arrangement of pores, size of pores, and porosity. The external pore dimensions were analyzed by optical microscope. Twenty-four pores, 4 in each of the 60 degrees range of the implants’ circumference, were randomly selected and the average dimension was calculated. Scanning electron microscopy was used to study the micro structural surface characteristics and pore morphology within the structure. The porosity and strut volume of the implant were determined using a helium-purged low-pressure pycnometer (Accelerated Surface Area and Porosity System, Micromeritics^®^ ASAP 2060 Pycnometer; Micromeritics Instruments Corporation, Norcross, GA, USA).

### 2.3. Evaluation of the Mechanical Properties of the Dental Implants

The stability (screw-in torque, screw-out torque, and pull-out strength) of the different DMLS dental implants was tested in synthetic cancellous bone block (Sawbones; Pacific Research Laboratories Inc., Vashon Island, WA, USA). Three-point bending tests were performed using an ElectroPuls^TM^ Instron E3000 (Norwood, MA, USA). Failure of the DMLS dental implants was evaluated using a torsional test machine (SE MODEL 2205NS; SE Test system Co., Ltd., Taipei, Taiwan) and NTS torsional load cells (NTS TECHNOLOGY Co., Ltd., Tokyo, Japan). 

### 2.4. In Vitro Cell Observation and Osteogenic Differentiation

#### 2.4.1. Scanning Electron Microscopy

Ti_6_Al_4_V discs (2.0 cm diameter × 0.5 cm height) resembling the dental implant surfaces with different porosities (0, 20, 40, 60 or 80%; 300–500 μm pore size) were produced using the same DMLS technique. The human MG63 osteosarcoma cell line was provided by American Type Culture Collection (ATCC, Rockville, MD, USA; obtained from Sigma Corp.: Sigma-Aldrich; St Louis, MO, USA). Confluent MG63 (ATCC, Rockville, MD, USA) osteosarcoma cell line cultures were seeded on top of the sterilized discs. The discs with MG63 cells were placed in a culture plate for 4 h for cell adhesion and then cultured in Dulbecco’s modified Eagle’s medium (Gibco, Life Technologies Inc., Grand Island, NY, USA). Fresh medium was replaced every 2 days. The morphology of cell proliferation was observed by field emission gun SEM (FEG-SEM) (JEOL, JSM-6330F, Tokyo, Japan). At 14 days, cells were collected and subjected to subsequent tests.

#### 2.4.2. Alkaline Phosphatase Level

Alkaline phosphatase (ALP) activity was analyzed with the SensoLyte^®^ pNPP Alkaline Phosphatase Assay Kit (AnaSpec, Inc., Fremont, CA, USA). Briefly, ALP in biological samples was detected by enzyme-linked immunoabsorbent assay with alkaline phosphatase conjugated secondary antibody or streptavidin using colorimetric pNPP (p-Nitrophenyl phosphate phosphatase) substrate. Upon dephosphorylation, pNPP turned yellow and was detected at absorbance = 405 nm. 

#### 2.4.3. Quantitative Real-Time Polymerase Chain Reaction (PCR)

Real-time PCR was performed with TaqMan^®^ Gene Expression Assays (Applied Biosystems, Foster City, CA, USA) according to manufacturer’s instructions, to determine the levels of the ALP and glyceraldehyde 3-phosphate dehydrogenase (GAPDH) mRNA transcripts. The expression levels of the ALP were calculated by normalizing the mRNA level against that of GAPDH as an internal control. 

### 2.5. In Vivo Biocompatibility Study

#### 2.5.1. Animal Experiments

The experimental protocols were approved by the Medical College’s Animal Research Committee of the National Taiwan University. Twenty-four adult (1-year-old) female New Zealand white rabbits were randomly divided into control (non-porous) and experimental (porous) groups. The dental implants were inserted into lateral aspect of distal femurs. Both groups were randomly subdivided into two subgroups (six animals each), representing euthanasia 6 and 12 weeks after implant insertion. 

#### 2.5.2. Micro-Computed Tomography (Micro-CT) Analysis

The dental implants after 6/12 weeks of implantation was examined by micro-CT (SkyScan 1176, Bruker, Kontich, Belgium) prior to pull-out test. The images of the bone and dental implants in the specimens were acquired and reconstructed by CTvox 2.4 software (Bruker Skyscan, Konitch, Belgium).

#### 2.5.3. Ex Vivo Mechanical Testing

An axial pull-out force was applied using a material testing machine (MTS 858, MTS Systems, Eden Prairie, MN, USA). Each implant was extracted from the distal femur at a rate of 5 mm/min until failure, which was defined as the maximum pull-out strength before the load decreased abruptly. 

### 2.6. Statistical Analysis

Results from mechanical experiments were expressed as mean ± standard deviation and statistically analyzed by two-way analysis of variance (ANOVA). Statistical significance by Dunnett’s test was set at *p* < 0.05. The unpaired two-tailed student’s *t*-test was performed to compare differences between the groups for in vitro and ex vivo experiments. Differences were considered significant at *p* < 0.05. All analyses were performed using SPSS version 16.0 software (SPSS Inc., Chicago, IL, USA).

## 3. Results

### 3.1. Gross and Microscopic Characterization

**Figure 1:**
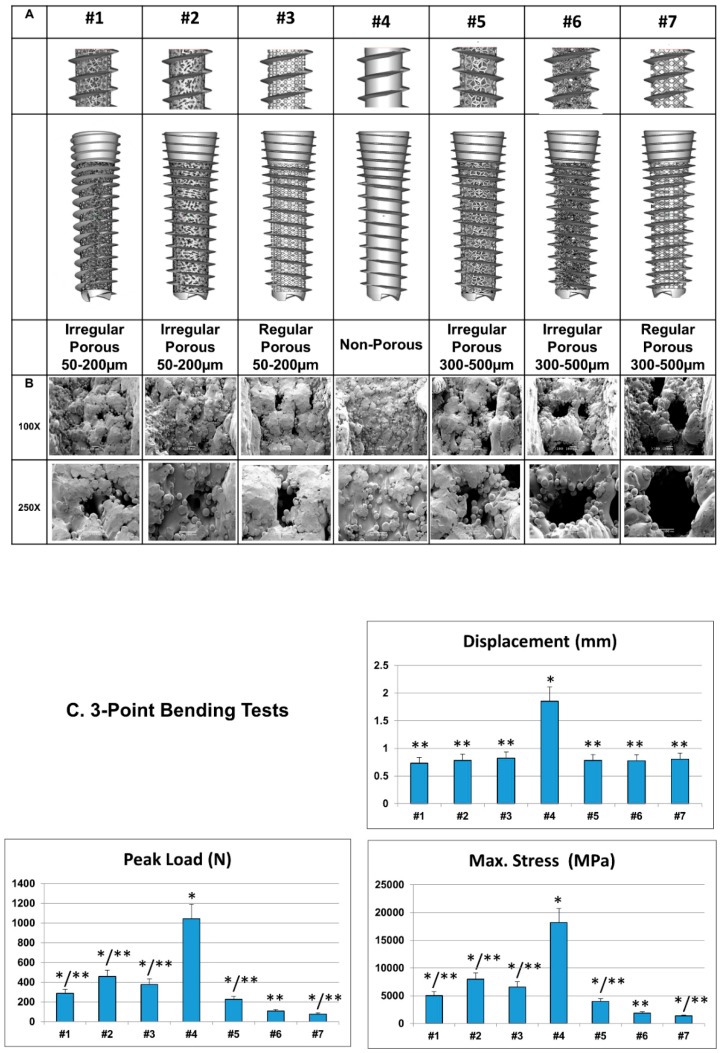
Gross morphology of the biomimetic direct metal laser sintering (DMLS) Ti_6_Al_4_V dental implants. (**A**) External characterization: the computer-assisted-design (CAD) of the external configurations of the seven different dental implants. (**B**) Scanning electron microscopy (SEM) images. SEM images showed no inter-layer difference when probed on the external portion of the implants. Although the surfaces of the implants were very rough, the struts were well formed and continuous. Bar = 100 µm. (**C**) Biomechanical parameters (3-point bending test) of the biomimetic Ti_6_Al_4_V dental implants. The non-porous design had superior biomechanical profiles compared to the porous designs. (n = 7 for each design. * indicates significant differences when compared to #6 dental implant; ** indicates significant differences when compared to #4 dental implant).

Figure 1A and Supplementary Table S1 show the configurations and biomechanical parameters of the seven different implants generated. There were four major characteristic parameters: arrangement of pores (regular versus irregular), strut volume (47.2–96.1 mm^3^), pore size (non-porous, 50–200 µm or 300–500 µm), and porosity (17–55%). SEM (Figure 1B) showed no inter-layer difference when probed on the exterior, indicating complete melting of powder metal and metallurgical bonding between layers during fabrication process. Although the surfaces of the implants were very rough, the struts were well formed and continuous.

### 3.2. Mechanical Properties

Results of the 3-point bending tests are shown in Figure 1C and Supplementary Table S1. The peak loads distributed between 78–1044 N, displacements at the peak load ranged from 0.73–1.85 mm, and the maximal stresses attained were 1358–18179 MPa. Flexural strength decreased significantly with the reduction of strut volume. Since all implant specimens had identical basic shapes, an increase in pore size or porosity reduced the total strut volume, resulting in the decreased effective stiffness and ultimate strength of the implant. For the porous scaffold dental implants, irregular arrangement of pores also appeared to result in better mechanical properties compared to regular arrangement.

**Figure 2:**
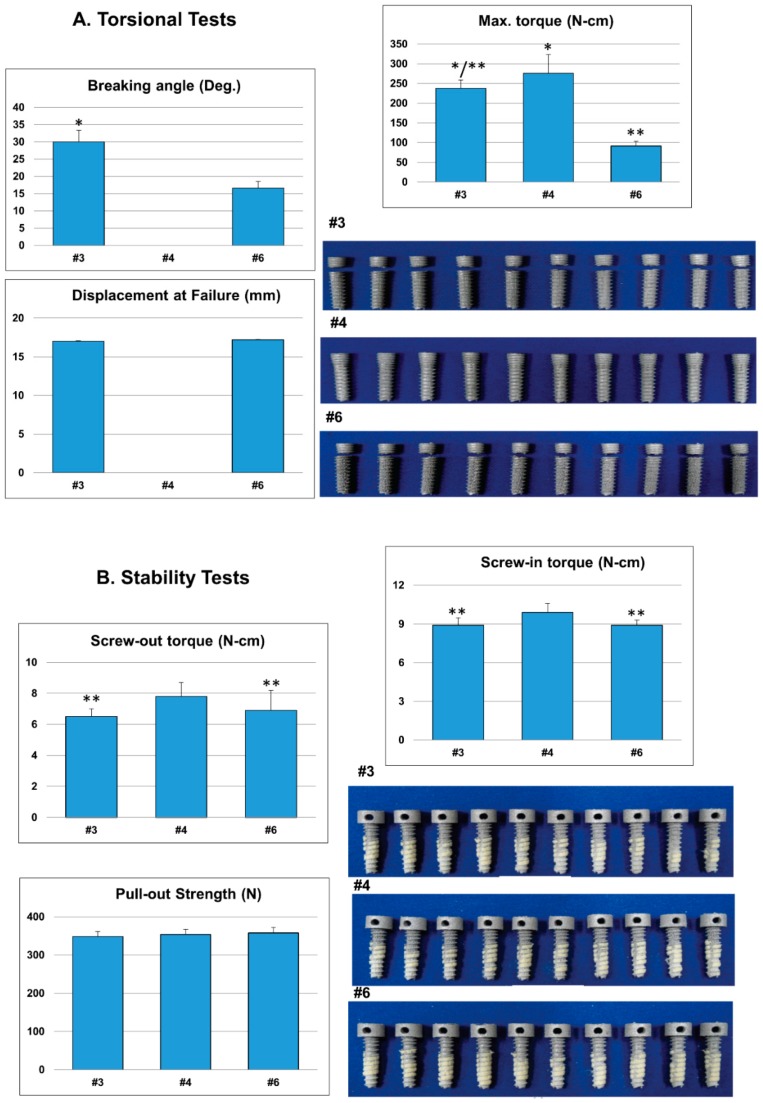
(**A**) Torsional and (**B**) stability tests of the biomimetic DMLS Ti_6_Al_4_V dental implants (n = 10). During torsional testing, the highest mean maximal torque value was obtained for the non-porous design. Throughout the entirety of the torsional experiments, type #4 dental implants (non-porous) did not break. During stability testing, the mean screw-in or screw-out torque was significantly higher for the non-porous design, while no significant differences existed in the pull-out strength among the three designs tested. (* indicates significant differences when compared to the #6 dental implant; ** indicates significant differences when compared to the #4 dental implant).

As the irregular arrangement of pores appeared to result in better mechanical properties than regular arrangement, and an increase in pore size or porosity resulted in the decreased mechanical properties of the implant, we choose irregular arrangement (with higher porosity), regular arrangement (with lower porosity) to minimize the configuration effect. Type #3 (regular, 50–200 µm pores, 36% porosity), #4 (non-porous), and #6 (irregular, 300–500 µm pores, 55% porosity) implants were selected for further biomechanical experiments. Results from torsional tests showed that the maximum torque was highest for the type #4 implant (276.0 ± 47.4 N-cm), followed by #3 (237.2 ± 21.5 N-cm), and least for the type #6 (91.5 ± 12.1 N-cm) (Figure 2A; Supplementary Table S2). Throughout torsional testing, the type #4 implant did not break. Results from the stability tests showed that the screw-in or screw-out torque was significantly higher with the type #4 implant (Figure 2B; Supplementary Table S2), while no significant difference was exhibited for pull-out strength among the three designs.

**Figure 3:**
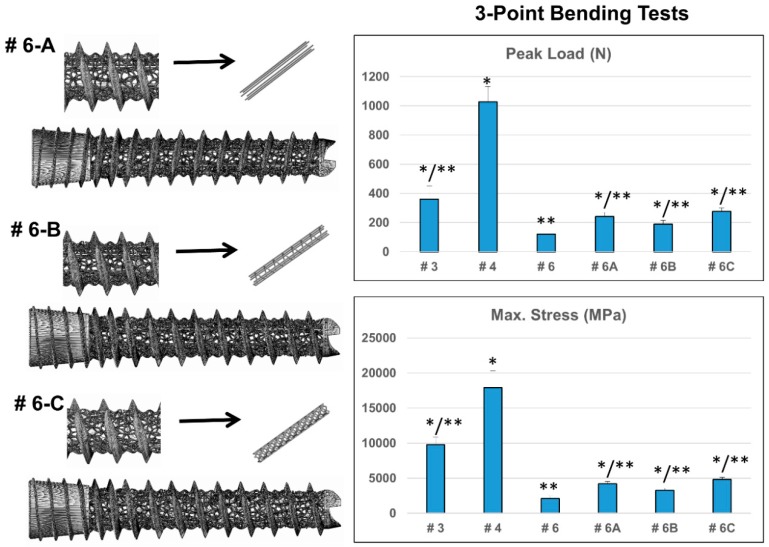
Morphological characteristics and biomechanical parameters of the augmented biomimetic DMLS Ti_6_Al_4_V dental implants (n = 10 for each design). The weaker type #6 dental implant (300–500 µm, 55% porosity) was augmented with three different designs of longitudinal printed strut to optimize its biomechanical characteristics. The resulting augmented dental implants (type #6-A, #6-B, and #6-C) all exhibited superior biomechanical profiles compared to the original design (#6). Approximately a 2.3 times increase in both peak load and maximal stress were observed for type #6-C implant. (* indicates significant differences when compared to the #6 dental implant; ** indicates significant differences when compared to the #4 dental implant).

Since the type #6 implant was designed with the larger pore size and highest porosity, it had the least strut volume and the weaker mechanical properties among the various designs. We further augmented type #6 implants with three different designs of longitudinal 3D-printed struts to improve the biomechanical characteristics (Figure 3). Irrespective of the strut designs, all the augmented dental implants (#6-A, #6-B, #6-C) exhibited superior biomechanical profiles compared to the original design (#6). Both the peak load and maximum stress increased approximately double the original value (Figure 3; Supplementary Table S3).

### 3.3. In Vitro and In Vivo Biocompatibility of the Biomimetic Ti_6_Al_4_V Implants

**Figure 4:**
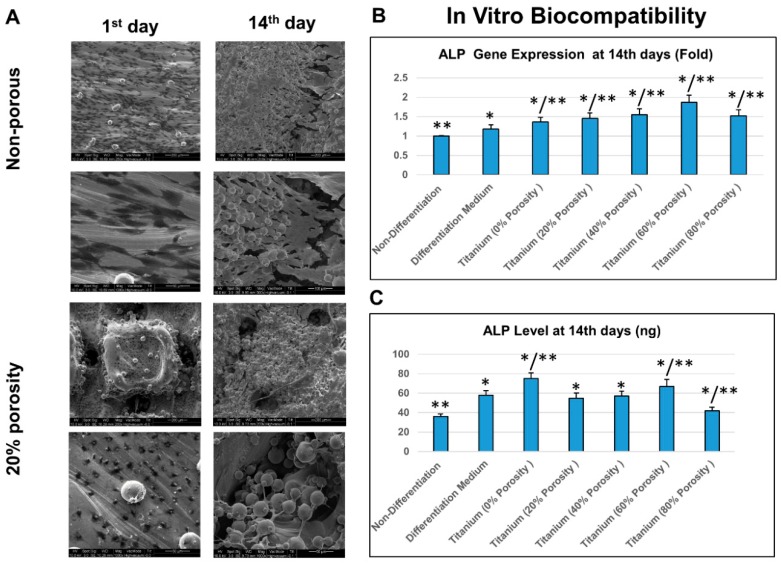
MG63 cells differentiation on DMLS Ti_6_Al_4_V discs: in vitro biocompatibility analysis. (**A**) MG63 cells attached, proliferated and differentiated well on both non-porous and porous DMLS Ti_6_Al_4_V discs. The MG63 cell clusters attached on the surface of discs and grew into the pores. Representative results of 10 independent experiments are shown. (Length of scale bar: as described) (**B**) Comparisons of alkaline phosphatase (ALP) mRNA gene expressions or (**C**) relative ALP activity after 14 days of culturing MG63 cells in control medium (non-differentiation), osteogenic (differentiation) medium, or on Ti_6_Al_4_V discs of various (0, 20, 40, 60 or 80%) porosities (n = 10). The ALP gene expression was significantly up-regulated in the titanium samples, which attained maximal expression at 60% porosity.

SEM showed that MG53 cells attached, proliferated, and differentiated well on all DMLS Ti_6_Al_4_V discs. The MG63 cell clusters attached on the surface of the discs and grew into the surface pores (Figure 4). The relative ALP mRNA expressions and ALP activities were significantly up-regulated when MG53 cells were co-cultured with Ti_6_Al_4_V discs for 14 days. ALP gene of cells grown on the 60% porosity discs achieved maximal expression.

**Figure 5:**
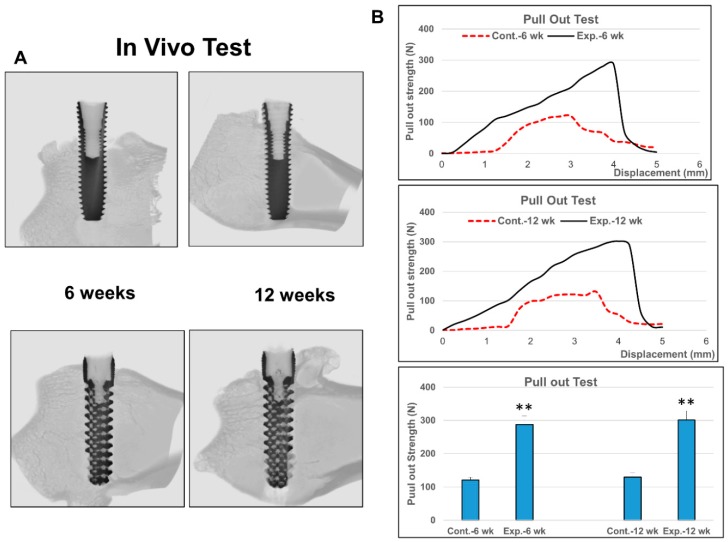
Biocompatibility and biomechanical parameters of the biomimetic DMLS Ti_6_Al_4_V dental implants in animal experiments (n = 6 in each group). (**A**) Micro-computed tomography analysis showed good incorporation of bone onto the threads of type #4 non-porous dental implant and into the porous scaffold structures of the type #6 dental implant. (**B**) Mechanical tests showed significantly higher pull-out strength (2.4 folds) for the type #6 porous dental implants at both 6 and 12 weeks. (** indicates significant differences when compared to #4 non-porous dental implant).

Animal study and micro-CT analysis showed good incorporation of bone onto the threads of type #4 non-porous DMLS dental implants, and into the porous scaffold structures of the type #6 implants (Figure 5). Mechanical tests showed that the pull-out strength of type #4 non-porous and type #6 porous implants were 121.4 N versus 287.5 N at 6 weeks, respectively, and 129.9 N versus 301.5 N at 12 weeks, respectively. 

## 4. Discussion

Scaffold design enhances biological anchorage to the surrounding bone by allowing ingrowth of mineralized tissue into the porous network and has been a significant feature in the application of orthopaedic tissue engineering [12]. Several methods have been proposed to produce porous metal structures [4,13]. More advanced techniques that have the capability of controlling the external and internal structure are additive manufacturing 3D-printing procedures including electron beam melting [14], selective laser melting [15], direct metal deposition [16], and selective laser sintering [17]. In this study, direct metal laser sintering of pre-alloyed Ti_6_AlV_4_ fine powder was used to additively manufacture the dental implants. The theoretical formulation assumes a block with an infinite number of pores. In our previous study on the interference screws, the surface roughness and inter-connected porous architectures enhanced better bone-tendon-implant integration and resulted in stronger biomechanical characteristics when compared to traditional screws [18]. However, during the manufacturing process, only a finite number of pores could be built into the implants. The differences between theoretical and experimentally observed values could be attributed to the melting of titanium alloy powder at high temperatures, and the subsequent solidification by cooling caused an unevenness of the surface, leading to pore surface curvatures and corrugation (Figure 1). While the overall porosity, the dimension of the solid structures, and pore size collectively determine the biomechanical properties of the dental implants, a linear correlation between porosity and strength does not seem to exist (Supplementary Table S1). This may be partly due to the fact that curvatures and corrugations of pore surface result in local heterogeneities that affect peak stress [19].

Although the exact optimal parameters have not been validated in literature, studies have revealed that 500 μm pore-sized titanium scaffolds are more beneficial for in vitro cells growth and in vivo bone formation than larger pores [20,21]. In general, the minimum requirement for pore size is ≈100 μm; however, pore sizes >300 μm are recommended to enhance new bone formation and the formation of capillaries [12,13]. A porosity of 60–70% could best fit the mechanical properties of human trabecular bone [22]. In this study, types #3 (50–200 µm, 36% porosity), #4 (non-porous), and #6 (300–500 µm, 55% porosity) dental implants were selected for further biomechanical tests. Although pull-out strengths were not significantly different among the three designs when tested in sawbones (Figure 2B), after 6 or 12 weeks of implantation into rabbit femurs, bone grew into the scaffold structure of the type #6 implant, and its pull-out strength increased to approximately 2.4-fold that of the non-porous implant (Figure 5). While relatively high porosity and large pore sizes attain greater bone ingrowth, they result in diminished mechanical properties. We further augmented the weakest #6 design with a 3D-printed longitudinal strut. The longitudinal strut acted like a weight-bearing beam and improved the biomechanical characteristics of the type #6 implant without significantly compromising the porosity (Figure 3; Supplementary Table S3). Addition of the localized struts reduced the overall porosity from 55% to 43–46%. However, the improvement of the mechanical properties was not due to a decrease of the porosity alone, as type #6-A (averaged 46% porosity) still demonstrated overwhelmingly better performance than the type #7 dental implant (generalized 47% porosity) (Supplementary Table S1). 

Very few clinical studies on DMLS dental implants were found in current literature [11]. These studies were conducted by the same group of researchers and the Ti_6_AlV_4_ dental implants were characterized by a superficial porous surface (non-scaffold) on a bulk and dense core [23]. Despite that the original intention of fabricating these implants was to avoid stress shielding by reducing elastic modulus, the Young’s moduli of the porous surface and compact core were 77 ± 3.5 GPa and 104 ± 7.7 GPa, respectively, still much higher than that of bone. Nevertheless, these clinical studies have reported satisfactory short-term results. Two animal studies evaluated the osseointegrative properties of titanium alloy dental implants with scaffold design [24,25]. In a sheep model, the DMLS implants were made with a solid core and 500 μm thick macroporous shell (pore diameter approximately 500 μm) [24]. In a rabbit model, multi-rooted selective laser melting implants were designed with 26% porosity (300 μm pores) in the cortical part and 50% porosity (400 μm pores) in the cancellous part [25]. Despite the completely different geometric characteristics of the implants, both studies showed significant improvement of removal torque values after 8 weeks of implantation. One recent study specifically investigated the optimal pore size (comparing 0, 200, 350 and 500 μm) for scaffold Ti_6_Al_4_V dental implants using 2D photoelastography on bone mimicking tissue phantom and in vitro experiments [26]. Results showed a lower stress shielding to the surrounding bone at the porous structure of the implants and that both 350 and 500 μm pore sizes presented a notable improvement in cell proliferation, attachment, and differentiation [26]. Taken together, the parameters used in our type #6 implant (300–500 µm, 55% porosity) seem to provide an optimal potential for improving the mechanical shielding to the surrounding bones and osteoinduction of the implant itself. However, the intraoral environment is constantly changing and dental implants always experience cyclic occlusal functioning. As implants are likely to fracture upon extended use, augmentation of the porous implants with 3D-printed longitudinal weight-bearing struts could be helpful. Additional studies will be necessary to validate this proposition. The idea of incorporating scaffold design into dental implants is emerging. Dental implants with porous tantalum components have been recently developed by Zimmer Inc. [27]. Tantalum and titanium both belong to the refractory metal group. Unlike titanium that is widely distributed and abundant on Earth, tantalum is rare and costly. In addition to the advantage of cost-saving, using DMLS technique to fabricate porous titanium alloy dental implants allows tailoring of the implants to accommodate the underlying anatomy of the individual patients. 

In conclusion, this study investigated the biomechanical and biological characteristics of scaffold Ti_6_Al_4_V dental implants manufactured using DMLS addition-production technology. Our results support that implants with interconnected pores encourage bone ingrowth and mineralization to achieve better retention. Based on our results so far, parameters such as pore size 300–500 µm and approximately 55% porosity could provide an optimal potential for improving the mechanical shielding effect and osseointegration, bearing in mind that these data were tested only on a standard dental implant shape of 4.3 mm in diameter and 10.0 mm in length. As an increase in pore size or porosity reduces total strut volume, the effective stiffness and ultimate strength of the implant also decrease. To achieve the ideal dental implant requirement of a combination of high strength and low elastic modulus, we introduced a new design concept that by augmenting the implant with a longitudinal weight-bearing strut, there is a chance to reach a balance of both biologic and mechanical demands. Future studies will be necessary to evaluate the fatigue behavior of these DMLS scaffold Ti_6_Al_4_V dental implants with/without augmented weight-bearing strut(s) possibly in a simulated oral environment to understand the long-term performance of these dental implants. 

## 5. Clinical Relevance

The Ti_6_Al_4_V dental implants manufactured using DMLS addition-production technology with interconnected pores (pore size 300–500 µm and 55% porosity) encourage bone ingrowth and provide an optimal potential for improving the mechanical shielding effect and osseointegration. As an increase in pore size or porosity reduces total strut volume, the effective stiffness and ultimate strength of the implant also decrease. We introduced a new design concept that by augmenting the implant with a longitudinal weight-bearing strut to achieve the ideal dental implant requirement of a combination of high strength and low elastic modulus, we are able to achieve a balance of both biological and mechanical demands, providing long-term performance of these dental implants.

## Supplementary Materials

The following are available online at http://www.mdpi.com/1996-1944/12/1/164/s1, Table S1: Morphological characteristics and biomechanical parameters (3-point bending test) of the biomimetic direct metal laser sintering (DMLS) Ti_6_Al_4_V dental implants (n = 7 for each design); Table S2: Torsional and stability tests of the biomimetic DMLS Ti_6_Al_4_V dental implants (n = 10; * indicates significant differences when compared to #6 dental implant; ** indicates significant differences when compared to #4 dental implant); Table S3: Three-point bending tests of the biomimetic DMLS Ti_6_Al_4_V dental implants (n = 10; * indicates significant differences when compared to #6 dental implant; ** indicates significant differences when compared to #4 dental implant).
