# Supplementary material for: Augmentation of DMLS Biomimetic Dental Implants with Weight-Bearing Strut to Balance of Biologic and Mechanical Demands: From Bench to Animal
Source: Materials (Basel). 2019 Jan 7;12(1):164. doi: 10.3390/ma12010164 (PMC6337105; doi:10.3390/ma12010164)
Supplement: Supplementary file 1 [file materials-12-00164-s001.pdf]

Supplementary

Augmentation of DMLS Biomimetic Dental Implants with Weight-Bearing Strut to Balance of Biologic and Mechanical Demands: From Bench to Animal

Jenny Zwei-Chieng Chang [1], Pei-I Tsai [2,3], Mark Yen-Ping Kuo [1,*], Jui-Sheng Sun [4,*], San-Yuan Chen [2] and Hsin-Hsin Shen [3]

1 School of Dentistry, College of Medicine, National Taiwan University, Taipei 10051, Taiwan; jennyzc@ms3.hinet.net
2 Department of Materials Science and Engineering, National Chiao-Tung University, Hsinchu 30010, Taiwan; peiyi@itri.org.tw (P.-I.T.); sanyuanchen@mail.nctu.edu.tw (S.-Y.C.)
3 Biomedical Technology and Device Research Laboratories, Industrial Technology Research Institute, Hsinchu 31040, Taiwan; shenhsin@itri.org.tw
4 Department of Orthopedic Surgery, College of Medicine, National Taiwan University, Taipei 10002, Taiwan
* Correspondence: oddie@ntu.edu.tw (M.Y.-P.K.); drjssun@gmail.com (J.-S.S.); Tel.: +886-2-23123456 (ext. 67083) (M.Y.-P.K.); +886-2-23224112 (J.-S.S.)

Received: 8 December 2018; Accepted: 27 December 2018; Published: 7 January 2019

Appendix Table 1. Morphological characteristics and biomechanical parameters (3-point bending test) of the biomimetic direct metal laser sintering (DMLS) Ti6Al4V dental implants (n = 7 for each design).

#	Characteristics	Volume	Pore Size	Porosity		Peak Load	Displacement	Max. Stress
		(mm^3)	(μm)			(N)	(mm)	(MPa)
#1	Irregular, Porous	69.9	50–200	0.34	Mean	288.0	0.73	5015.0
					SD	57.6	0.16	1053.2
#2	Irregular, Porous	87.6	50–200	0.17	Mean	458.7	0.78	7987.0
					SD	68.8	0.14	1517.5
#3	Regular, Porous	67.3	50–200	0.36	Mean	378.0	0.82	6582.0
					SD	83.2	0.20	1184.8
#4	Non-Porous	96.1		0	Mean	1044.0	1.85	18179.0
					SD	156.6	0.43	3454.0
#5	Irregular, Porous	72.1	300–500	0.31	Mean	227.0	0.78	3952.0
					SD	51.5	0.15	897.1
#6	Irregular, Porous	47.2	300–500	0.55	Mean	107.0	0.78	1863.0
					SD	20.2	0.15	395.0
#7	Regular Porous	56.5	300–500	0.47	Mean	78.0	0.80	1358.0
					SD	15.8	0.19	294.7

Appendix Table 2. Torsional and stability tests of the biomimetic DMLS Ti_6Al_4V dental implants (n = 10; * indicates significant differences when compared to #6 dental implant; ** indicates significant differences when compared to the #4 dental implant).

TORSIONAL TEST	#3	#4	#6	*p* Value (ANOVA)
Maximum torque (N-Cm)	237.2 ± 21.5*/**	276.0 ± 47.4*	91.5 ± 12.1**	<0.001
Breaking angle	30.0 ±	N/A	16.6 ±	

(Deg.)	3.4 *		1.9	
Displacement at Failure (mm)	17.0 ± 0.06	N/A	17.2 ± 0.05	
**STABILITY TEST**				
Screw-in Torque (N-cm)	8.9 ± 0.6**	9.9 ± 0.7	8.9 ± 0.4**	<0.001
Screw-out Torque (N-Cm)	6.5 ± 0.5**	7.8 ± 0.9	6.9 ± 1.3**	<0.001
Pull-out Strength (N)	348.6 ± 13.0	353.8 ± 13.6	357.6 ± 14.6	0.59

Appendix Table 3. Three-point bending tests of the biomimetic DMLS Ti_6Al_4V dental implants (n = 10; * indicates significant differences when compared to the #6 dental implant; ** indicates significant differences when compared to the #4 dental implant).

	**#3**	**#4**	**#6**	# **6-A**	# **6-B**	# **6-C**	***p* Value (ANOVA)**
**Porosity**	0.36	0	0.55	0.46	0.44	0.43	
**Peak Load (N)**	361.6 ± 89.6	1028.3 ± 104.6	120.5 ± 24.0	241.3 ± 28.2	187.9 ± 27.3	276.6 ± 23.4	<0.001
**P Value**	*/**	*	**	*/**	*/**	*/**	
**Max. Stress (MPa)**	6778.7 ± 1070.3	17905.0 ± 2414.5	2097.3 ± 374.1	4202.0 ± 327.7	3272.0 ± 268.3	4816.5 ± 314.2	<0.001
***p* Value**	*/**	*	**	*/**	*/**	*/**	
